# Benjamin Felson (1913-1988): A Pioneer of Modern Chest Radiology

**DOI:** 10.7759/cureus.117255

**Published:** 2026-09-30

**Authors:** Sohan Kundu, Aditya R Kundur, Padma Anne

**Affiliations:** 1 Health Sciences, Collin College, Frisco, USA; 2 Radiology, Texas Health Dallas, Frisco, USA

**Keywords:** biographies, historical vignette, historical vignettes, medical innovation, medical stories

## Abstract

Benjamin Felson was one of America's most influential chest radiologists, whose work reshaped how physicians teach and interpret chest radiographs. Felson spent nearly the entirety of his 50-year academic career at the University of Cincinnati, where he trained generations of physicians in radiographic interpretation. Before the advent of CT scanning, Felson named and popularized anatomy-based diagnostic signs, including the silhouette sign and the air bronchogram sign, giving practitioners a systematic basis for localizing and interpreting chest disease. His textbook, *Fundamentals of Chest Roentgenology* (1960), remains in continuous publication more than six decades later. The journal he founded in 1966, *Seminars in Roentgenology*, established the single-topic issue format later adopted across clinical medicine. This review discusses the life of Benjamin Felson, his diagnostic contributions, and his lasting legacy in radiology.

## Introduction and background

In 1895, Wilhelm Conrad Röntgen discovered X-rays and laid the foundation for radiology, which became a vital tool in medicine. Soon after, imaging of the respiratory system developed quickly in North America during the spring of 1896 [1]. Pioneers such as Francis H. Williams showed how chest imaging could identify devastating diseases, such as tuberculosis, pneumonia, and pneumothorax. Williams had accumulated over 100 volumes of tracings of clinical chest fluoroscopy, and with his clinical experience and dedication to patients' well-being, many clinical observations were made within the first few years of the discovery of X-rays. A few key advances followed soon after, including the "densitometer" (standardized measurements of gradual X-ray attenuation in the lungs) and the discovery that tuberculosis could be detected by fluoroscopy [1]. Chest radiography rapidly gained clinical adoption.

As chest radiography became capable of detecting an increasing number of diseases, interpreting these radiographs grew more complex for physicians. An extensive knowledge of human anatomy was required, along with the ability to recognize patterns associated with different diseases. As radiology continued to expand, there was a growing need for more organized methods of image interpretation and education. While early pioneers such as Williams had established the clinical value of chest imaging, later educators such as Benjamin Felson would help trainees and practicing physicians interpret the growing volume of radiographic information [1].

This article examines the life and contributions of Benjamin Felson, a chest radiologist who spent nearly his entire academic career at the University of Cincinnati (Figure 1) [2] and reshaped how physicians read and interpret chest radiographs. Felson worked during a period when the chest film was the primary imaging tool available for evaluating heart and lung disease. Computed tomography (CT), magnetic resonance imaging (MRI), and most modern cross-sectional imaging did not exist for the majority of his career. Reading a chest radiograph well was therefore a critical clinical skill, and one that was difficult to teach systematically.

**Figure 1:**
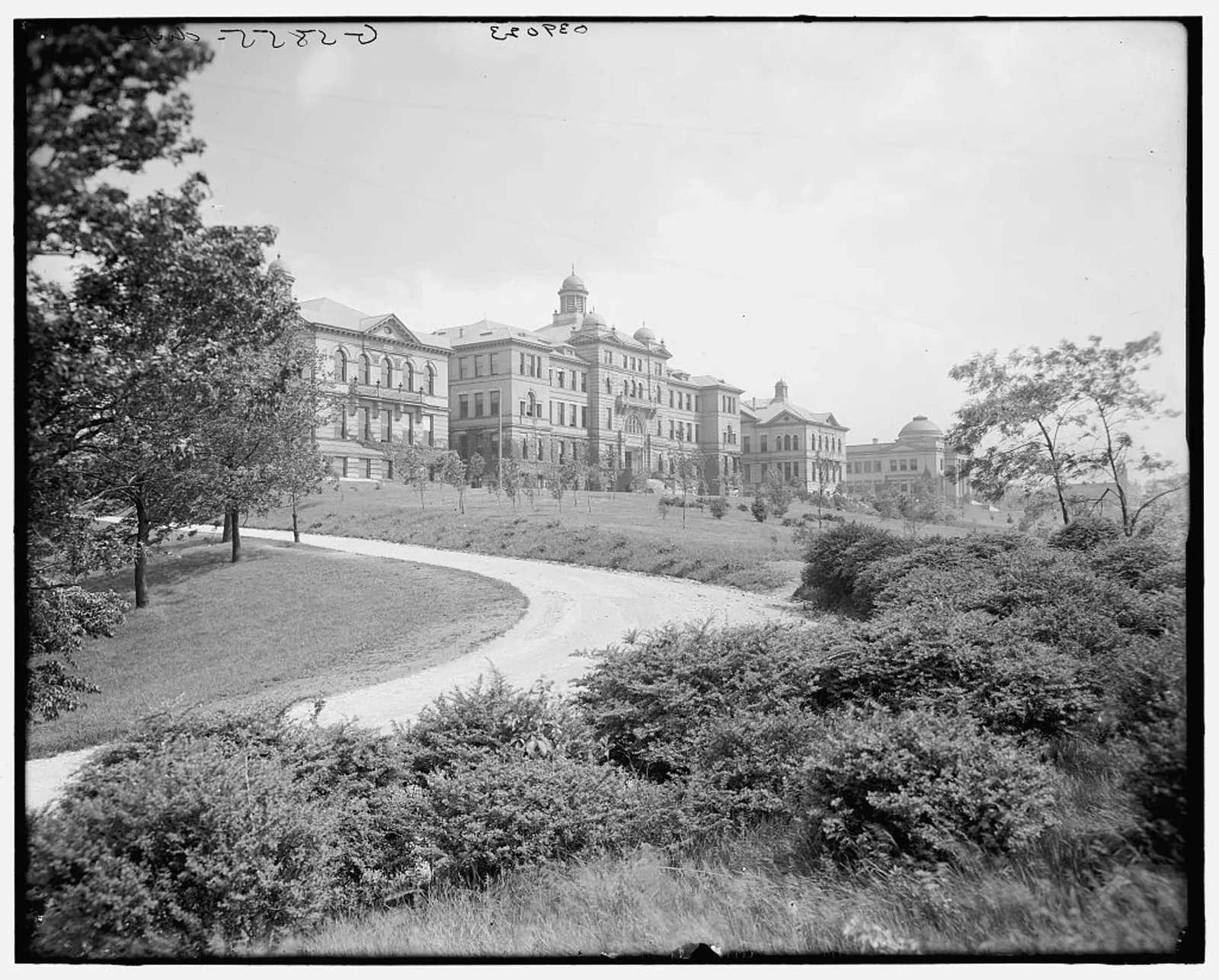
University of Cincinnati, Burnet Woods, Cincinnati, Ohio (circa 1900-1915) Credit: Detroit Publishing Co., Public Domain, via Library of Congress Prints & Photographs Division [3]

Felson addressed this challenge with a set of simple, anatomy-based signs, particularly the silhouette sign, which he named and popularized, giving physicians a logical framework for localizing disease on a chest radiograph [4]. These signs are still used daily in hospitals around the world [5]. His textbook has been in continuous publication for more than 60 years [6]. The journal he founded in 1966 continues to be published today [7]. His name endures through the endowed chair at Cincinnati established in his honor in 1987, one year before his death [2].

These frameworks worked by teaching physicians to recognize specific, describable patterns on a chest radiograph rather than relying on memorization of individual disease presentations. By translating years of clinical experience into named, repeatable rules, Felson made a highly technical skill accessible to trainees at every level, and his approach continues to shape how chest radiographs are taught and interpreted today [5,6].

## Review

Education and career

Benjamin Felson was born in Newport, Kentucky, in 1913, to Solomon "Sol" Felson (1873-1934) and Esther Bussel (Busel) Felson (1881-1941) [8]. His father was born in Dvinsk, Russia [8]. Solomon had an impoverished childhood and eventually immigrated to the United States in 1902, where he established a tailor shop in Newport [8]. The shop had a reputation for being a hub for political discussions, which led Solomon to become an active member of the socialist Workmen's Circle [6,8].

Felson's mother was born in Lyakhovichi, Russia (now Belarus) and later moved to Warsaw, where she met Solomon [8]. They married in 1901 and had a family of 10 children [8].

Felson later moved to Cincinnati, Ohio, with his family, where he grew up and attended the University of Cincinnati for both his undergraduate education and medical school. He earned his Doctor of Medicine in 1935 [2]. After completing an internship and a residency in pathology along with radiology at Cincinnati General Hospital, Felson completed additional training through a cancer therapy fellowship at Indianapolis City Hospital [7].

Medicine ran in the family: his brother Henry Felson (1907-1998) became an internist, and his brother Walter Felson (1908-1980) practiced as a family physician in Ohio [6].

In 1937, he married Virginia Raphaelson, and they had five children: Steve, Nancy, Marcus, Richie, and Eddie [6]. Colleagues described Felson as being as warm and generous in his personal life as he was rigorous in his professional one [6,9].

Military service

In 1942, Felson joined the United States Army Medical Corps and served as Chief of Radiology at the 28th General Hospital in Europe [7].

After leaving the army as a major in 1945, he returned to Cincinnati and joined the faculty of the University of Cincinnati College of Medicine, as well as the medical staff of Cincinnati General Hospital (Figure 2) [7]. He also maintained ties to the military and federal medical systems throughout his career, serving as a consultant to the US Air Force, the Walter Reed Army Medical Center, the Armed Forces Institute of Pathology, and, later, a panel of consultants to the National Institute for Occupational Safety and Health [7].

**Figure 2:**
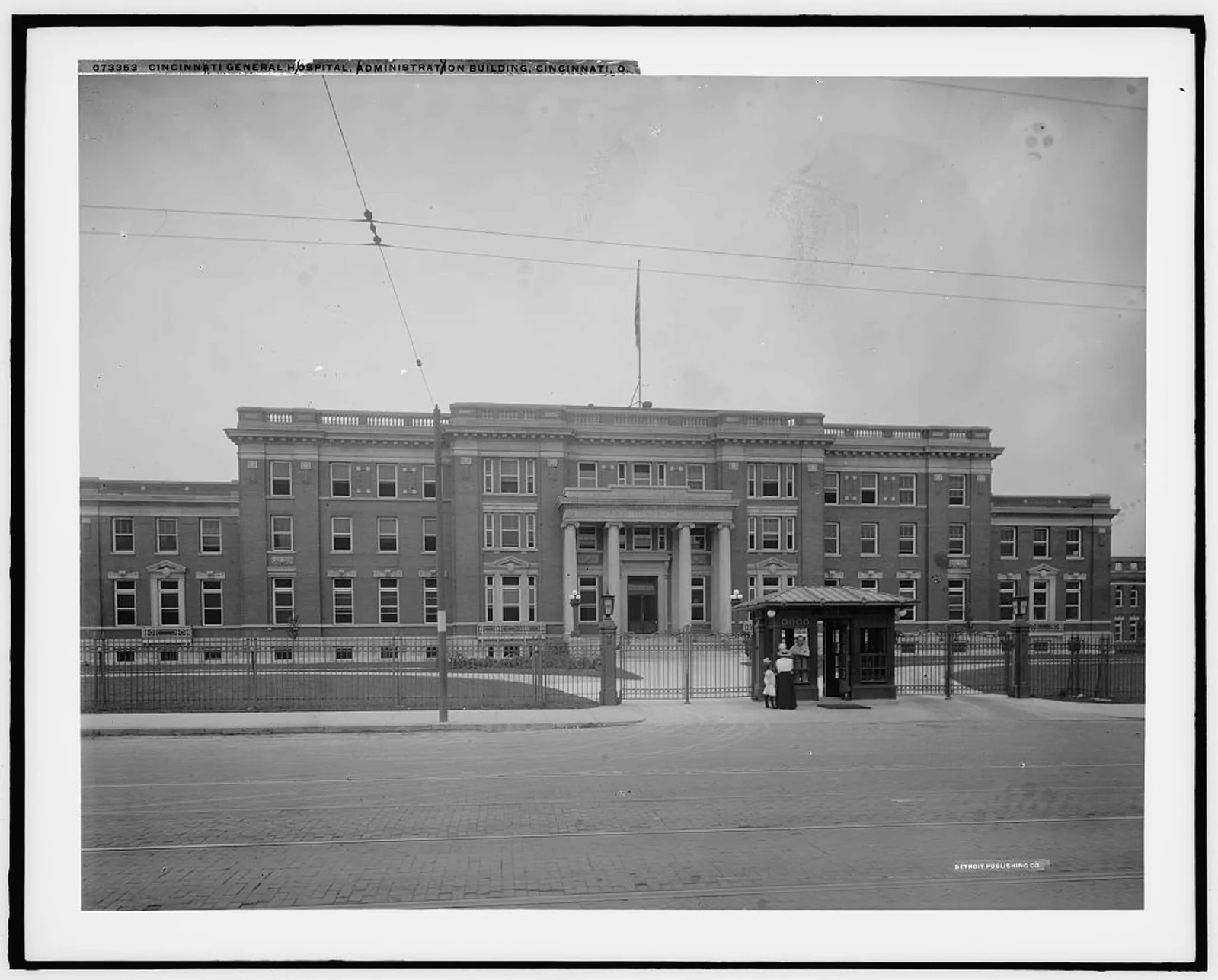
Cincinnati General Hospital, administration building, Cincinnati, Ohio (circa 1910-1920), where Felson directed the Department of Radiology from 1951 to 1973 Credit: Detroit Publishing Co., Public Domain, via Library of Congress Prints & Photographs Division [10]

Academic career at the University of Cincinnati

Felson returned to Cincinnati in 1945 and joined the Department of Radiology at Cincinnati General Hospital as an Assistant Professor. He was promoted to Associate Professor in 1948 and to full Professor in 1951, the same year he was appointed Director of the Department of Radiology, a position he held for 22 years, until 1973 [2,11]. He remained on the faculty as Professor until 1983 and then as Professor Emeritus until his death in 1988. In total, he gave the University of Cincinnati 50 years of service [2,11].

Under Felson's direction, the radiology training program at Cincinnati General Hospital grew into one of the largest in the country, and his teaching was honored eight times with the University of Cincinnati College of Medicine's Golden Apple Award [7,12]. His teaching reach extended past Cincinnati as well: Felson traveled internationally to help build radiology education in countries where the specialty was still developing [7]. He also served as First Vice President of the Radiological Society of North America (RSNA), chaired the RSNA Commission on Education, and was a member of the Board of the American College of Radiology (ACR) [7].

Felson died suddenly of a heart attack on October 22, 1988, while at work on a manuscript [9]. He was succeeded as the leading figure of the department by his trainee and colleague, Dr. Jerome H. Wiot [12].

Diagnostic contributions

The Silhouette Sign

The silhouette sign, popularized by Felson, is one of the most influential signs used in radiologic diagnosis [4]. The basis of the silhouette sign is that the border between two neighboring structures is only visible if their densities are different. For instance, on a chest radiograph, the border between the lungs filled with air and soft tissue organs such as the heart is usually well defined because air and soft tissue attenuate X-rays differently. However, when fluid, pus, blood, or other cellular matter accumulates in the lungs, the affected region assumes a radiographic opacity similar to that of the neighboring organ of soft tissue. As a result, the border between the two neighboring structures becomes blurred or even invisible, forming the silhouette sign [4].

Felson and his brother, Henry Felson, MD, an internist, published and named this observation "the silhouette sign" in their 1950 paper in Radiology; the term was later popularized more broadly through his 1960 textbook [6]. Felson and Henry acknowledged Dunham's earlier description while introducing and popularizing the term "silhouette sign" in the medical literature.

The sign is not without limitations, and later work has qualified some of the original teaching. The sign depends on anatomic contiguity between a lesion and a border-forming structure, so it can be falsely negative when a lesion lies close to, but does not actually touch, that border, and falsely positive when volume loss, prior surgery, or radiotherapy has altered the expected anatomic relationships [5]. The 2014 re-examination of Felson's signs using modern CT correlation confirmed the sign's continued diagnostic value but also clarified several of these edge cases and showed how cross-sectional imaging can resolve ambiguity in cases where the plain-film sign alone is equivocal [5]. These refinements do not diminish the sign's teaching value, but they underscore that it functions best as a starting point for localization rather than a standalone diagnostic rule.

The Air Bronchogram Sign

The air bronchogram sign, incorporated by Benjamin Felson into his systematic framework for chest radiograph interpretation, assists radiologists in identifying diseases affecting the lungs [5,6]. Air-filled bronchi become radiolucent branching structures within surrounding air-space opacity when the adjacent lung fills with fluid or inflammatory material. The air bronchogram sign is an important diagnostic clue when dealing with conditions such as pneumonia, pulmonary edema, and other diseases affecting the lung parenchyma [5].

Mediastinal Localization Signs

One of Benjamin Felson's most significant contributions to chest radiology was the compartment-based system he articulated for mediastinal localization, described in his own 1969 paper (Figure 3) [13]. The mediastinum is the central compartment of the thoracic cavity located between the lungs. This region consists of numerous important structures, including the heart, trachea, esophagus, and major blood vessels. Diagnosis is essential in determining the location of an abnormality because of the sheer number of diseases that occur in this visceral compartment. In the early years of radiology, many physicians often struggled to identify the origin of mediastinal masses using radiographs alone. Felson addressed this challenge by developing a system that divided the mediastinum into anatomical compartments: the anterior, middle, and posterior [13]. Using this, radiologists could narrow their range of possible diagnoses. Nowadays, mediastinal compartment-based localization assists physicians in further guiding advanced imaging through CT, MRI, or positron emission tomography (PET) scans.

**Figure 3:**
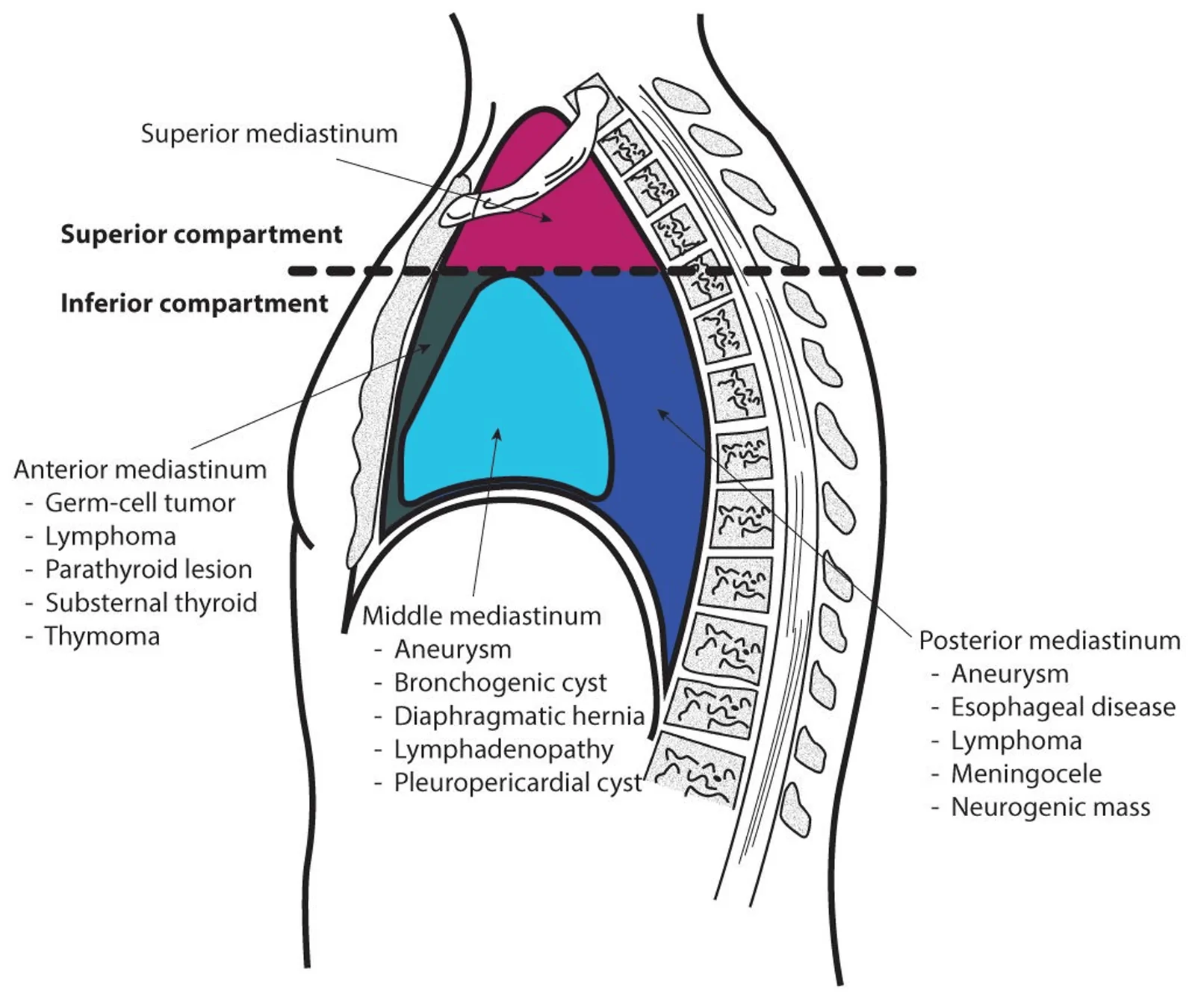
Mediastinal compartments (superior, anterior, middle, and posterior) shown in sagittal view, with representative pathologies characteristic of each region Credit: B Parker, CC BY-NC-ND 4.0, via StatPearls [14]

The "Aunt Minnie" Concept

The "Aunt Minnie" concept is among Felson's most influential teachings [6,9]. The idea is based on a simple analogy: if a person walks down the street looking, dressing, and behaving exactly like one's Aunt Minnie, there is no need to ask for identification. Similarly, if a radiographic image displays all the characteristics of a well-known disease, then physicians can recognize the diagnosis immediately. Felson used this concept to help teach his students pattern recognition skills associated with specific diseases. However, Felson underscored the importance of clinical judgment when findings are atypical. Therefore, this skill allows physicians to effectively identify common diseases and develop expertise [6].

Scholarly works and publications

Fundamentals of Chest Roentgenology (1960) and Its Successors

Felson's most influential work, *Fundamentals of Chest Roentgenology*, was first published in 1960 and quickly became a standard reference for interpreting chest radiographs [6]. The book was distinctive for its programmed-learning format, walking readers through a structured sequence of questions and images rather than presenting the material as a conventional narrative text [15]. This approach let physicians test their own pattern recognition as they read, reinforcing the same systematic thinking that underlies the silhouette sign and Felson's other diagnostic frameworks. Successive editions preserved the original programmed-text format while updating the imaging technology discussed, and the book has since been retitled *Felson's Principles of Chest Roentgenology*, now in its sixth edition [15]. *Seminars in Roentgenology*

In 1966, Felson founded *Seminars in Roentgenology*, a journal organized on issues revolving around a single topic rather than a collection of unrelated papers [7]. Each issue covered one subspecialty topic in depth, functioning more like a curated review monograph than a traditional journal. This format was an early and influential adoption of the single-topic issue model in medical publishing, and proved highly effective for specialty education. Felson edited the journal and contributed the well-known "Letter from the Editor," which readers valued for combining genuine teaching with a light touch [7].

Gamuts in Radiology (1975)

In 1975, Felson co-edited the first edition of *Gamuts in Radiology: Comprehensive Lists of Roentgen Differential Diagnosis* with Maurice Reeder [6]. A "gamut" is an organized list of reported causes of a given radiographic finding. The concept addressed a genuine clinical problem: once a radiologist identifies a plausible diagnosis, it is easy to stop searching. Providing a structured list of every known cause of a given finding compels a more thorough review. The book became an immediately popular reference, and Reeder continued the series after Felson's death, expanding it into subspecialty volumes covering neurological, cardiovascular, and musculoskeletal radiology [6].

In total, Felson published more than 150 peer-reviewed papers spanning pulmonary infections, lung cancer, occupational lung disease, and cardiovascular imaging (Table 1) [2,6,7,11].

**Table 1:** Timeline of major milestones in the career of Benjamin Felson ACR: American College of Radiology; RSNA: Radiological Society of North America

Year	Milestone
1913	Born in Newport, Kentucky
1935	Earned Doctor of Medicine, University of Cincinnati
1942	Joined the U.S. Army Medical Corps during World War II
1945	Returned to Cincinnati; joined the faculty as Assistant Professor
1950	Published the silhouette sign with Henry Felson
1951	Appointed Director, Department of Radiology, Cincinnati General Hospital
1959	Served as First Vice President of the RSNA
1960	Published *Fundamentals of Chest Roentgenology*
1966	Founded *Seminars in Roentgenology*
1969	Published “The mediastinum,” describing mediastinal localization signs
1973	Stepped down as Director, Department of Radiology
1975	Co-edited *Gamuts in Radiology* with Maurice Reeder
1979	Awarded the RSNA Gold Medal
1985	Designated a Living Legend in Radiology
1987	Benjamin Felson Chair of Radiology established at Cincinnati
1988	Awarded the ACR Gold Medal; died on October 22

Awards and recognition

Benjamin Felson's dedication to medical education and research earned him numerous honors from prestigious organizations in the field of radiology [7]. Among his most notable achievements was the Gold Medal of the RSNA, which he received in 1979 [16]. The RSNA Gold Medal is the organization's highest distinction and is awarded for exceptional contributions to the science of radiology [16]. By the time he received this honor, Felson had transformed chest radiology through his teaching methods and publications, most notably *Fundamentals of Chest Roentgenology* (1960). His work had already established a systematic approach to interpreting radiographs and influenced physicians from around the world. Later in 1988, Felson was awarded the Gold Medal of the ACR, one of the most prestigious recognitions in American radiology [7].

Internationally, Felson was one of only a few American radiologists elected an Honorary Fellow of the Royal College of Radiologists of Great Britain [7]. In 1985, Felson was designated the title of a Living Legend in Radiology [7]. In further tribute, an annual Felson Lectureship was established in Felson's honor at Tel Aviv University in Israel [9]. Separately, the University of Cincinnati established the Benjamin Felson Chair of Radiology in 1987 [2].

Felson as a teacher

Felson is considered to be one of the most impactful teachers of his era, not only in radiology but in medicine [6]. His colleague Maurice Reeder described him as the "greatest radiologist of his time," noting that more than anyone else, Felson had enhanced the reputation and knowledge of the specialty through his inquisitiveness and his gift for communication with both the written and spoken word [6].

Another close colleague, Dr. Harold Jacobson, wrote that Felson's impact on medical students was legendary: no student, whether radiologist or not, who had attended one of his lectures could forget the experience [9]. Felson taught physicians from all backgrounds how to read a chest radiograph well, and his textbooks, journal, and lectures all improved one's practical knowledge [7].

Felson was also well known for his humor. His 1989 book *Humor in Medicine and Other Topics* collected essays he had written over the years. Felson maintained that clarity and scientific rigor were not mutually exclusive. In his textbook, he wove anecdotes into the clinical teaching, contributing to the text's sustained use as a reference for more than six decades [6].

Felson's continuing legacy

The chest radiograph is the most commonly performed imaging study in the world [17,18]. Felson's teachings of the silhouette sign, the air bronchogram sign, and other principles remain widely used in introductory chest radiography education. Physicians can read the sixth edition of *Felson's Principles of Chest Roentgenology*, which was published in 2026 [15]. Additionally, a 2014 review article in the *Journal of Medical Imaging and Radiation Oncology*, titled "Felson Signs Revisited," illustrated each of his signs using modern CT images [5].

The signs have remained relevant because they are rooted in anatomy rather than any single imaging technology. The underlying reasoning applies across analogue film and digital radiography, both of which rely on the same two-dimensional projection principles the signs were designed for. Cross-sectional modalities such as CT visualize the same anatomy directly and can resolve cases where plain-film signs are ambiguous, but they do not simply reproduce the original signs; rather, they complement them by removing the superimposition the signs were designed to interpret.

Felson's approach to education with his systematic teaching works has influenced a generation of radiology educators and continues to do so. The programmed-learning format of his textbook anticipated later evidence-based teaching principles, including active recall and spaced repetition, which are now considered essential in medical education [19]. The thematic journal format he pioneered with *Seminars in Roentgenology* has since been adopted across numerous clinical specialties. Felson's influence, in this way, extended well beyond radiology into medical education more broadly.

## Conclusions

Benjamin Felson transformed chest radiography from a highly specialized skill into a systematic, teachable discipline. Through frameworks such as the silhouette sign, the air bronchogram sign, and mediastinal localization, he gave physicians a shared, anatomy-based vocabulary for recognizing disease on a chest radiograph. His influence extended well beyond his own publications: as an educator, journal founder, and mentor, he shaped how generations of radiologists were trained to interpret and communicate radiographic findings.

Felson's teaching principles continue to underpin how physicians approach the chest radiograph today, decades after his death. His career illustrates how clear, structured teaching can outlast the specific technology it was built around, and his legacy offers a lasting model for translating complex diagnostic reasoning into frameworks that remain accessible to new generations of clinicians.
